# They receive antenatal care in health facilities, yet do not deliver there: predictors of health facility delivery by women in rural Ghana

**DOI:** 10.1186/s12884-018-1749-6

**Published:** 2018-05-03

**Authors:** Michael Boah, Abraham B. Mahama, Emmanuel A. Ayamga

**Affiliations:** 1 Ghana Health Service, Kassena Nankana West District Health Directorate, P. O. Box 21, Paga, Upper East Region Ghana; 2 UNICEF- Ghana Country Office, Accra, Ghana

**Keywords:** Predictors, Ghana, Health facility, Delivery place, Skilled attendant

## Abstract

**Background:**

Research has shown that use of antenatal services by pregnant women and delivery in health facilities with skilled birth attendants contribute to better delivery outcomes. However, a gap exists in Ghana between the use of antenatal care provided by health facilities and delivery in health facilities with skilled birth attendants by pregnant women. This study sought to identify the predictors of health facility delivery by women in a rural district in Ghana.

**Methods:**

This was a cross-sectional study conducted in June 2016. Women who delivered in the past 6 months preceding the study were interviewed. Data on socio-demographic characteristics, use of antenatal care, place of delivery and reasons for home delivery were collected from study participants. Chi-square test and multiple logistic regression analysis were used to assess an association between women’s socio-demographic and obstetric characteristics and place of delivery at 95% confidence interval.

**Results:**

The study found that 98.8% of women received antenatal care services at least once during their recent pregnancy, and 67.9% attended antenatal care at least four times before delivery. However, 61.9% of the women delivered in a health facility with a skilled attendant. The frequently mentioned reason for home delivery was “unaware of onset of labour and delivery”. The odds for delivery at a health facility were reduced among women with four living children [(AOR = 0.07, CI = 0.15–0.36, *p* = 0.001)], with no exposure to delivery care information [(AOR = 0.06, CI = 0.01–0.34, *p* = 0.002), who started their first ANC visit from the second trimester of pregnancy[(AOR = 0.003, CI = 0.01–0.15, *p* < 0.001)] and increased among women who made at least four ANC visits before delivery [(AOR = 17.53, CI = 6.89–44.61, *p* < 0.001)].

**Conclusion:**

Findings from this study revealed a low rate of delivery at health facilities although visits to antenatal care sessions were high, an indication that there was the need to intensify health education on early initiation of antenatal care, signs of labour and delivery, and importance of health facility delivery.

## Background

Low-and middle-income countries (LMIC) contribute about 99% of the maternal deaths that occur worldwide [1]. An analysis of the causes of maternal deaths in Africa shows that most of these deaths are as a result of direct obstetric complications notably, haemorrhage, hypertension, sepsis and obstructed labour that emerge around the time of labour and delivery [2]. However, deaths from these complications can be prevented. In Ghana, though there has been a decline in maternal mortality from 760 deaths per 100,00 live births in 1990 to 319 deaths per 100,000 live births in 2015, it is still very high [1].

Complications that may arise from pregnancy are impossible to predict. The World Health Organization (WHO) therefore recommends delivery by a skilled birth attendant (SBA) [3]. The presence of an SBA at delivery has also been stressed in many summits and international conferences worldwide [4]. The contribution of skilled delivery in the reduction of deaths among pregnant women and neonates has been highlighted by several studies [5–7].

A high proportion of women in developing countries are reported to have received antenatal care (ANC) at least once from a qualified health provider during pregnancy [8–12]. However, in spite of the importance placed on the use of an SBA by women during delivery to prevent complications, it is not uncommon to find one in three women using an SBA during delivery, especially in sub-Sahara Africa [13]. Cost, number of ANC visits made by a woman before delivery, maternal education, poor attitude of health care providers, parity and distance, the effect of which is compounded by the lack of transport, have been identified as some barriers to the use of health facility by pregnant women for delivery in developing countries [14–17].

To improve geographic access to health services by all, the Community-based Health Planning and Services (CHPS) initiative was implemented by the Ghana Health Service (GHS) in 2002 and covered all the regions in Ghana. This was followed by the implementation of the free maternal health care policy in 2003 under which pregnant women do not pay user fees to receive health services from the time of pregnancy to 3 months postpartum. Additionally, routine health education and promotion activities on the benefits of delivery with a SBA are carried out [18, 19]. Consequently, nine out of ten women in Ghana have utilized ANC services from health professionals during their recent pregnancy [20–23]. However, the high prevalence of ANC usage does not translate into the use of health facility for delivery. The 2014 Ghana Demographic and Health Survey (GDHS) report revealed that an overwhelming 97% of women received ANC at least once during their recent pregnancy, but 73% went on to deliver in a health facility where SBAs are available [24].

With these strategies in place, it is expected that the high prevalence of ANC utilization should correspond to an increase in health facility delivery. This is however not the case. The question is why? The reasons for this phenomenon needed to be ascertained.

Skilled delivery in the Upper East Region of northern Ghana is high (84.1%), though there are variations across the thirteen districts in the region [24, 25]. The 2014 annual report of the Builsa district showed that an appreciable 71.5% of pregnant women received ANC services once during pregnancy, 90.7% made at least four follow up visits before delivery while only 46.2% delivered in a health facility [25, 26]. These findings on ANC utilization and health facility delivery put together demonstrate that a gap exists between receiving ANC services from health facilities and delivery in health facilities.

Studies conducted in Ghana [23, 27, 28] have identified factors such as maternal age, religion, household income and maternal occupation to predict health facility delivery. However, the focus of these studies still presents gaps in the literature. For instance, two of the studies did not assess the effect of the gestation of pregnancy at the onset of ANC on the place of delivery [23, 27]. Furthermore, the study by Esena & Sappor [28] was carried out in an urban setting, hence the results may not be wholly applicable in a rural setting. The gestation of pregnancy at the onset of ANC and place of residence (rural or urban) have been reported to influence the use of health facility for delivery services elsewhere [15, 20]. Therefore, results of this present study will inform policy and add to existing literature by examining the factors behind the existing gap between the use of ANC and health facility delivery by women.

The aim of this study was to explore socio-demographic and obstetric factors such as maternal age, education, ethnicity, religion, occupation, health insurance status, the gestation of pregnancy at first ANC attendance, and parity as predictors of health facility delivery by women in a rural district in northern Ghana.

## Methods

### Study setting

This study was cross-sectional by design. It was carried out in June 2016 in the Builsa South District, a predominantly rural district located in the Upper East Region of northern Ghana. The district comprises of six sub-districts. The district has 3 health centres and 14 CHPS zones serving a population of 38, 298 (projected from the 2010 population and housing census). Women in reproductive age form 24% (9192) of the total population of the district. There are no private maternity homes [26].

### Study population and sampling

Women included in this study were all women aged 15–49 years resident in the district who delivered in the past 6 months preceding the survey. Antenatal care record book of each study participant was used to confirm obstetric information given. Women not resident in the study district, who delivered more than 6 months preceding the survey were excluded.

A sample size of 423 women was computed using the formula, z^2^pq/d^2^: p, the proportion of first trimester ANC registrants was estimated as 49% from a previous study in Ghana [29] with 5% error margin at 95% confidence interval and 10% non-response rate.

A multi-stage sampling technique was used in the selection of study communities and participants. First, simple random sampling by lottery was used to select six communities, one in each of the six sub-districts. The total sample size was then allocated proportionally to the selected communities.

Second, a sampling frame of eligible women was developed with the aid of existing community filariasis and child welfare clinic registers in the health facilities serving the selected communities. The two registers were used in order to have an up-to-date information about the women in the various households. A systematic random sampling method was employed to select a participating household. Only one woman in participating household was interviewed. A lottery was used to select one woman for the interview whenever a selected household had more than one eligible woman.

### Variables

The delivery place was selected as the main outcome variable. It was dichotomized as 0 “Home” for all deliveries occurring outside the health facility and 1 “Health facility” for deliveries occurring in the health facility. This information was obtained by asking the question “Where did you deliver [name of child]?” Based on available literature, predictor variables included were age, marital status, occupation, highest educational level, religion, parity, ethnicity, health insurance validity status, exposure to information on delivery care (irrespective of source), the gestation of pregnancy at the onset of ANC and the number of ANC visits made before delivery.

### Data collection

Data on socio-demographic characteristics of respondents, place of delivery and reasons for home delivery were gathered using a structured questionnaire. The questionnaires were administered mainly in the local language (Buili).

Ten data collectors with at least secondary education and familiar with the customs and traditions of the area were recruited to assist in data collection. Six supervisors with previous experience in data collection supervised the data collection process. Data collectors and supervisors were trained together in a two-day training session. Training for the first day covered translation of the questionnaire from English to Buili and the interview process. A pre-test was carried out the second day in a neighbouring district with similar characteristics as the study district. Data collected in pre-test were excluded in the final analysis. Woman’s ANC card and index child’s road to health record booklet were used to confirm information given by mother where applicable.

### Data analysis

Administered questionnaires were checked daily for completeness and consistency. Data were entered in EpiData Entry Client version 2.06.20 (EpiData Association, Denmark), and the dataset exported to STATA 13 Special Edition (StataCorp, College Station Taxes, USA LP) for cleaning and analysis.

Pearson chi-square (χ^2^) and Fisher’s exact tests were used, where appropriate to identify associations between the predictor variables and the outcome variable. Tests were two-tailed and a *p* < 0.05 was considered statistically significant. A possible correlation between statistically significant variables was checked using the Pearson Correlation Matrix before they were put in a multiple logistic regression model. None was found to be collinear.

### Ethical considerations

Approval to carry out this study was obtained from the Ethics Review Committee of the Ghana Health Service (ID No. GHS-ERC 31/12/15). Participants consented by signing or thumb printing the consent form after the purpose and benefits of participating in the study were explained to them. Approval was obtained from parents of participants younger than 18 years before the interview. Anonymity and safety of participants were ensured.

## Results

### Socio-demographic characteristics of study participants

The mean age of participants was 29.2 ± 7.4 SD (range 15–49 years). The majority were married 402 (95.0%), were farmers 274 (64.8%) and belonged to the Christian religion 263 (62.2%). Of the 423 respondents, 172 (40.7%) had no formal education, 101 (23.9%) had two living children, 351 (83%) had ever enrolled onto the national health insurance scheme, and 254 (72.0%) had valid insurance during their recent pregnancy (see Table 1).

**Table 1:** Socio-demographic characteristics of the study participants (*N* = 423 unless stated)

Variable	Number	Percent
Age group (years)		
15–19	32	7.6
20–24	99	23.4
25–29	100	23.6
30–34	79	18.7
35–39	67	15.8
40 and above	46	10.9
Marital status		
Single	8	1.9
Married	402	95.0
Cohabiting	4	0.9
Separated	5	1.2
Widow	4	0.9
Highest educational level attained		
No education	172	40.7
Primary	137	32.4
Middle/Junior High School	67	15.8
Secondary and above	47	11.1
Occupation		
Unemployed	79	18.7
Famer	274	64.8
Self-employed	62	14.7
Formal sector employee	8	1.9
Religion		
Traditional	115	27.2
Christian	263	62.2
Muslim	45	10.6
Parity (Birth order)		
1	87	20.6
2	101	23.9
3	73	17.3
4	86	20.3
5+	76	18.0
Ethnic group		
Builsa	413	97.6
Mamprusi	4	0.9
Kassena	4	0.9
Sissala	2	0.5
Health insurance enrolment status		
Enrolled	351	83.0
Never enrolled	72	17.0
Health insurance status during recent pregnancy (*N* = 356)		
Valid	254	72.0
Not valid	97	28.0
Exposure to information on delivery care		
Yes	387	91.5
No	36	8.5
Type of information received (multiple responses allowed) (*N* = 387)		
Sleep under an insecticide-treated bed net	194	49.6
Observe personal and environmental hygiene	134	34.6
Deliver in health facilities	132	34.1
Practice exclusive breastfeeding for first 6 months	121	31.3
Send children for monthly growth monitoring and promotion	64	16.5
Signs of labour and delivery	35	9.0

### Obstetric characteristics of study participants

The obstetric characteristics of the study participants are presented in Table 2. Of the 423 women interviewed, 98.8% had attended antenatal care at least once during their recent pregnancy, and the majority (67.9%) made at least four visits before delivery. However, less than half (43.1%) started ANC in the first 3 months of their pregnancy. Many of the women received ANC services from the CHPS facility (60.3%) and health centres (38.5%). Regarding the place of delivery, 61.9% of respondents reported delivering in a health facility, of which the majority (84.4%) were assisted by the Midwife. The most frequently mentioned reasons for home delivery were unaware of onset of labour and delivery (68.0%), and no previous complications with delivery from previous pregnancies (19.6%).

**Table 2:** Obstetric characteristics of study participants (*N* = 423 unless stated)

Variable	Number	Percent
ANC use during recent pregnancy		
Yes	418	98.8
No	5	1.2
Gestation of pregnancy at first ANC visit (*N* = 418)		
First trimester	180	43.1
Second trimester	235	56.2
Third trimester	3	0.7
Source of ANC services for last pregnancy (N = 418)		
Hospital	1	0.2
Health centre/clinic	161	38.5
CHPS only	252	60.3
TBA only	1	0.2
CHPS & TBA	1	0.2
Number of ANC visits made before delivery (N = 418)		
1	1	0.2
2	14	3.3
3	119	28.5
4 and more visits	284	67.9
Place of delivery		
Health facility	262	61.9
Home	161	38.1
Assistance at health facility delivery (*N* = 262)		
Doctor	25	9.5
Midwife	220	84.0
CHN	17	6.5
^a^Reasons for home delivery (*N* = 161)		
Unaware of onset of labour and delivery	109	67.7
No complications with delivery from previous pregnancies	32	19.9
The home was the only delivery place available	18	11.2
Better care outside the health facility	8	5.0
Husband’s decision	6	3.7
Cheaper cost of delivery	4	2.5
Advice from mother-in-law	3	1.9
Health facility too far	2	1.2
No means of transport	1	0.6

^a^Multiple responses allowed

### Factors associated with place of delivery

A bivariate analysis was done to assess the association between individual socio-demographic and obstetric characteristics and the place of delivery. The results showed that nine of the eleven selected variables had a significant (*p* < 0.05) association with place of delivery. These variables included age, occupation, highest educational level attained, religion, parity, having a valid insurance during pregnancy, gestational age of pregnancy at the onset of ANC attendance, number of ANC visits made before delivery, and exposure to information on delivery care during the recent pregnancy (see Table 3).

**Table 3:** Factors associated with place of delivery in rural Ghana (*N* = 431 unless stated)

Exposure variable	n	Place of delivery		*P*-value
		Home	Health facility	
		n (%)	n (%)	
Age (years)				< 0.001
15–19	32	5 (15.6)	27 (84.4)	
20–24	99	20 (20.2)	79 (79.0)	
25–29	100	37 (37.0)	63 (63.0)	
30–34	79	33 (41.8	46 (58.2)	
35–39	67	36 (53.7)	31 (46.3)	
40 and above	46	30 (65.2)	16 (34.8)	
Marital status				0.921^a^
Single	8	2 (25.0)	6 (75.0)	
Married	402	154 (38.3)	248 (61.7)	
Cohabiting	4	1 (25.0)	3 (75.0)	
Separated	5	2 (40.0)	3 (60.0)	
Widow	4	2 (50.0)	2 (50.0)	
Occupation				< 0.001
Unemployed	79	49 (62.0)	30 (38.0)	
Farmer	274	90 (32.9)	184 (67.1)	
Self-employed	62	21 (33.9)	41 (66.1)	
Formal sector employee	8	1 (12.5)	7 (87.5)	
Highest educational level attained				< 0.001
No education	172	107 (62.2)	65 (37.8)	
Primary	137	42 (30.7)	95 (69.3)	
Middle/Junior High	67	8 (11.9)	59 (88.1)	
Secondary and above	47	4 (8.5)	43 (91.5)	
Religion				< 0.001
Traditional	115	70 (60.9)	45 (39.1)	
Christian	263	76 (28.9)	187 (71.1)	
Moslem	45	15 (33.3)	30 (66.7)	
Ethnicity				0.487^a^
Builsa	413	158 (38.3)	255 (61.7)	
Mamprusi	4	0 (0.0)	4 (100.0)	
Kassena	4	2 (50.0)	2 (50.0)	
Sissala	2	1 (50.0)	1 (50.0)	
Parity				< 0.001
1	87	12 (13.8)	75 (86.2)	
2	101	32 (31.7)	69 (68.3)	
3	73	23 (31.5)	50 (68.49)	
4	86	40 (46.5)	46 (53.5)	
5+	76	54 (71.1)	22 (28.9)	
Health insurance status during recent pregnancy (*N* = 351)				< 0.001
Invalid insurance	97	51 (52.6)	46 (47.4)	
Valid insurance	254	65 (25.6)	189 (74.4)	
Exposure to information on delivery care				0.001
Yes	387	138 (35.7)	249 (64.3)	
No	36	23 (63.9)	13 (36.1)	
Gestation of pregnancy at the onset of ANC (N = 418)				< 0.001
First trimester	180	7 (3.9)	173 (96.1)	
Second trimester and beyond	238	149 (62.6)	89 (37.4)	
Number of ANC visit made before delivery (*N* = 418)				< 0.001
Less than 4 visits	134	115 (85.8)	19 (14.2)	
4 and more visits	284	41 (14.4)	243 (85.6)	

^a^Fisher’s exact test

### Predictors of health facility delivery

Factors that were significantly associated with place of delivery in the bivariate analysis were put into a multiple logistic regression model to measure their strength of association. Parity, exposure to information on delivery care, the gestation of pregnancy at the onset of ANC, and the number of ANC visits made before delivery were strong predictors of delivery in a health facility (see Table 4). The odds of delivering in a health facility decreased with increasing parity from two children to four living children. Compared to women with one living child, women with two, three or four living children had 77.0%, 80.0% and 93.0% less the chances of delivering in health facility respectively [(AOR = 0.23, CI = 0.06–0.90, *p* = 0.034), (AOR = 0.20, CI = 0.04–0.99, *p* = 0.048), and (AOR = 0.07, CI = 0.15–0.36, *p* = 0.001) respectively]. However, women with at least five living children had increased odds of delivering in a health facility compared with women with four living children [(AOR = 0.11, CI = 0.02–0.67, *P* = 0.017)]. Also, women without exposure to information on delivery during their recent pregnancy were 94% less likely to deliver in a health facility [(AOR = 0.06, CI = 0.01–0.34, *p* = 0.001)]. Additionally, women who started their first ANC visit from the fourth month of pregnancy had 97.0% fewer chances of delivering in a health facility compared to women who started ANC in the first 3 months of pregnancy [(AOR = 0.03, CI = 0.01–0.15, *p* < 0.001)]. Furthermore, the odds of using a health facility for delivery were about 18 times more among women who attended ANC for at least four times before delivery compared to women who made less than four ANC visits before delivery [(AOR = 17.53, CI = 6.89–44.611, *p* < 0.001)].

**Table 4:** Predictors of health facility delivery in rural Ghana

Exposure variable	AOR(95% CI)	P-value
Age (years)		
15–19	1.00	
20–24	1.67 (0.26–10.60)	0.586
25–29	1.34 (0.20–8.83)	0.76
30–34	1.64 (0.22–12.42)	0.631
35–39	0.95 (0.11–7.90)	0.959
40 and above	0.58 (0.06–6.08)	0.654
Occupation		
Unemployed	1.00	
Farmer	7.88 (2.45–25.33)	0.001
Self-employed	5.13 (1.39–18.87)	0.014
Formal sector employee	5.97 (0.44–80.94)	0.178
Highest educational level attained		
No education	1.00	
Primary	1.10 (0.42–2.84)	0.848
Middle/Junior High	0.44 (0.09–2.21)	0.318
Secondary and above	0.32 (0.04–2.56)	0.284
Religion		
Traditional	1.00	
Christian	0.80 (0.31–2.07)	0.651
Moslem	5.96 (1.59–22.41)	0.008
Parity		
1	1.00	
2	0.23 (0.06–0.90)	0.034
3	0.20 (0.04–0.99)	0.048
4	0.07 (0.15–0.36)	0.001
5+	0.11 (0.02–0.67)	0.017
Health insurance status		
Invalid insurance	1.00	
Valid insurance	1.50 (0.63–3.58)	0.363
Exposure to information on delivery care		
Yes	1	
No	0.06 (0.01–0.34)	0.002
Gestation of pregnancy at the onset of ANC		
First trimester	1.00	
Second trimester and beyond	0.03 (0.01–0.15)	< 0.001
Number of ANC visit made before delivery		
Less than 4 visits	1.00	
4 and more visits	17.53 (6.89–44.61)	< 0.001

## Discussion

The results from this study aimed at identifying the predictors of health facility delivery in a rural district in Ghana showed that ANC utilization is high in the study area, as 98.8% of women reported using ANC at least once during their recent pregnancy and about 68.0% made four and more visits before delivery. However, the proportion of women who delivered in a health facility was 61.9% which was not very different from the proportion of women who made at least four follow up visits before delivery (67.9%). The high ANC utilization and low health facility delivery in this study conform to findings from other studies conducted in Ghana [20, 22, 23, 30] and other parts of sub-Saharan Africa [31–33]. The “unaware of onset of labour and delivery” mentioned as the main reason for home delivery in this study possibly explains the gap that exists between ANC utilization and health facility delivery. Women may be more vulnerable during labour than during ANC. Therefore, in the case of ANC utilization, they may have the privilege of time in deciding when, how and where they want to use ANC service but the situation is different when labour starts. The situation may be exacerbated when the woman does not know or recognize early the signs of labour and delivery.

Factors such as age, occupation, maternal education, religion, parity, having a health insurance during pregnancy, exposure to information on delivery care during the recent pregnancy, gestational age of pregnancy at onset of ANC attendance and the number of ANC visits made before delivery were significantly associated with the place of delivery in the bivariate analysis. Older women (35 years and above) were more likely than younger women to deliver at home in this study. This may be because, after many previous deliveries, older women may build more self-confidence and experience in the childbearing process than younger women especially in the absence of any previous birth complications and may not see the relevance to deliver in the presence of a SBA. Also, older women may be deeply buried in traditional beliefs and practices that prohibit the uptake of modern medicine [34]. This could possibly explain the reason why women belonging to the traditional religious group were less likely than their counterparts to use a health facility for delivery as revealed in this study. Age and religion were positively associated with health facility delivery in a different district in the same region [34]. Having some source of income could imply an improvement in household socioeconomic status. Though delivery services are free in public health facilities in Ghana, there may be some indirect costs involved with the use of a health facility for delivery services such as the cost of transportation which is a barrier to the use of health facility for delivery by especially rural women [35] and other materials that may be required for delivery. An employed woman may be able to overcome this barrier easily relative to an unemployed woman. An educated woman relative to a woman with no formal education may have a better access to health-related information and be more concerned about her health and the health of her unborn baby thereby choosing to deliver in a health facility. High maternal education has been associated with increased use of maternal health services in Uganda [33] and Ethiopia [36]. The health insurance scheme in Ghana aims at removing out-of-pocket user fees as barriers to accessing health services [19]. In this study, having a valid health insurance was associated with health facility delivery. This finding supports the findings of another study in Ghana that reported high utilization of health facility delivery with the possession of a health insurance [37].

Findings from the study revealed that a woman with one child was more likely to use a health facility for delivery compared to a woman with two to four children. Also, a woman with five or more children was likely to deliver in a health facility compared with a woman with four children. It can be explained that a woman delivering for the first time does not have prior experience with childbirth, may lack self-confidence and consider the delivery very challenging and anticipate unforeseen complications. These may compel her to use a skilled attendant for a safer delivery. However, a woman delivering for the second, third or fourth time has had an experience with childbirth from her previous delivery. This may contribute significantly to her decision-making process as to whether to deliver at home or at a health facility, especially if she did not experience any complications with her previous delivery (ies). A study in Nigeria [38] reported that women were more likely to use maternal health services if they had a previous complication with delivery (e.g. stillbirth). Similar to a woman with one child, a woman with five and more children may consider herself at high risk of birth complications due to the many deliveries and thus recourse to a health facility for delivery where she is sure skilled attendants can manage any resulting complication. She may also be advised by health workers to deliver in a health facility because of the high risk involved [39]. A qualitative study in Africa reported that women perceived themselves to be at risk of adverse pregnancy outcomes after five pregnancies [40]. The positive association between parity one and health facility delivery has also been reported by studies conducted in Senegal, Zambia and Kenya [41–43]. However, a study in Nigeria did not report any association between parity and delivery in a health facility [8].

This study identified that women who were not exposed to information on delivery care during their recent pregnancy were less likely to use the health facility for delivery when compared to women who were exposed to information on delivery during their recent pregnancy. The knowledge of women regarding the benefits of health facility delivery and the opportunities available for life-saving in case of any negative outcomes may be enhanced through exposure to information on delivery care. As explained, the perceived benefits of delivery in a health facility may be decided by the knowledge of the mother on the dangers associated with home delivery and life-saving opportunities available in health facilities [44]. Another study in northern Ghana also reported a positive association between media exposure and health facility delivery [30]. A study in Ethiopia reported that women who were exposed to information on maternal health relative to women who did not receive information were over eight times more likely to give birth in a health facility [45]. Though exposure to maternal health information is very instrumental in promoting health-seeking behaviour, the type of information received should also be taken into serious consideration. Although 91.5% of the women in this study received maternal health information during their recent pregnancy, the frequently mentioned (49.6%) health information received by women was about sleeping under insecticide-treated bed net while 9.0% of the women mentioned that they received information on signs of labour and delivery. This might have contributed to the reason (unaware of onset of labour and delivery) for home deliveries in this study mentioned earlier.

Furthermore, this study found that women who initiated ANC in the first trimester of pregnancy and women who made at least four ANC follow up visits before delivery had increased odds of using a health facility for delivery. Starting ANC in within the first 3 months may offer the woman the opportunity to attend many times and have more contact with health professionals before delivery. These contacts, first of all, serve as a window of opportunity for health professionals to provide the woman with adequate information on the benefits derived from delivering in a health facility and the dangers associated with home delivery. This might enhance the woman’s knowledge on maternal health and promote delivery in a health facility. A study in Kenya concluded that starting ANC in the first trimester was a precursor for delivery in a health facility [46]. Similarly, a study in Mali reported that women whose knowledge on maternal health was enhanced through contacts with health staff during ANC were more likely to deliver in health facilities [47]. Secondly, a woman may build confidence and trust in health professionals from continuous visits to the health facility for ANC during pregnancy. She may also listen to encouragement and advice from these health workers to deliver in the health facility. Other studies have also reported that at least four ANC visits before delivery increases the woman’s chances of using the health facility for delivery [34, 43, 48, 49].

This study, like many other studies, has strengths and limitations. The major strength of this study was that it limited respondents to women who delivered in the past 6 months, thereby minimizing the tendency of recall bias. Also, obstetric history of the woman was confirmed from the woman’s ANC card to ensure the credibility of obstetric information obtained. However, a limitation of this study was its design as cross-sectional. A cross-sectional study only assesses association and not causality. Additionally, the study did not assess the association between non-use of ANC and delivery place.

However, in spite of these limitations, the findings from this study provide information on predictors of health facility delivery in the study district since it is the first of its kind, and adds to existing literature in Ghana on issues related to maternal health-seeking behaviour.

## Conclusions

This study sought to identify the predictors of health facility delivery in a rural district in Ghana. Factors such as maternal age, occupation, the educational level attained, religion, parity, health insurance status, exposure to information on delivery care, the gestation of pregnancy at the start of first ANC visit and the number of ANC visits made before delivery were more likely to influence the choice of delivery place. Additionally, the small proportion of women who received health information on signs of labour and delivery might have contributed to the significant proportion of home deliveries in this study since the common reason cited for home delivery was “unaware of onset of labour and delivery”.

In addition to using the socio-demographic and obstetric characteristics of the women by health care professionals (especially midwives) to identify potential women who will attend ANC and still deliver at home, there is an urgent need to intensify health education especially during focused ANC sessions on signs of labour and delivery, and birth preparedness to enhance the knowledge of women to be able to identify early signs of labour and report to the nearest health facility to deliver with SBAs.

## Abbreviations

ANC: Antenatal care,
CHPS: Community Health Planning and Services,
DDHS: District Director of Health Service,
GDHS: Ghana Demographic and Health Survey,
GHS: Ghana Health Services,
LMIC: Low and middle-income country,
RDHS: Regional Director of Health Service,
SBA: Skilled birth attendant,
WHO: World Health Organization
